# Puma predation on radiocollared and uncollared bighorn sheep

**DOI:** 10.1186/1756-0500-2-230

**Published:** 2009-11-18

**Authors:** Sean M Clemenza, Esther S Rubin, Christine K Johnson, Randall A Botta, Walter M Boyce

**Affiliations:** 1 Wildlife Health Center, University of California, Davis, CA, USA; 2 Conservation Biology Institute, Borrego Springs, CA, USA; 3 California Department of Fish and Game, San Diego, CA, USA

## Abstract

**Background:**

We used Global Positioning System (GPS) data from radiocollared pumas (*Puma concolor*) to identify kill sites of pumas preying upon an endangered population of bighorn sheep (*Ovis canadensis*) in southern California. Our aims were to test whether or not pumas selected radiocollared versus uncollared bighorn sheep, and to identify patterns of movement before, during, and after kills.

**Findings:**

Three pumas killed 23 bighorn sheep over the course of the study, but they did not preferentially prey on marked (radiocollared) versus unmarked bighorn sheep. Predation occurred primarily during crepuscular and nighttime hours, and 22 kill sites were identified by the occurrence of 2 or more consecutive puma GPS locations (a cluster) within 200 m of each other at 1900, 0000, and 0600 h.

**Conclusion:**

We tested the "conspicuous individual hypothesis" and found that there was no difference in puma predation upon radiocollared and uncollared bighorn sheep. Pumas tended to move long distances before and after kills, but their movement patterns immediately post-kill were much more restricted. Researchers can exploit this behaviour to identify puma kill sites and investigate prey selection by designing studies that detect puma locations that are spatially clustered between dusk and dawn.

## Background

Pumas (*Puma concolor*) are known predators of bighorn sheep (*Ovis canadensis*) in North America, but puma behaviour and movements associated with these predation events are poorly understood. Ross et al. [1] found predation on Rocky Mountain bighorn sheep to be an individual behaviour in Alberta, and Logan and Sweanor [2] and Ernest et al. [3] also presented evidence for differences in the frequency that individual pumas killed desert bighorn sheep in the southwestern United States. Although these studies identified individual pumas that selectively killed bighorn sheep, they left important questions unanswered. During ongoing studies of pumas and endangered bighorn sheep in the Peninsular Ranges of California, we radiocollared 3 pumas (1 female and her 2 offspring) who subsequently each killed multiple bighorn sheep (total ≥ 23). This gave us the opportunity to critically evaluate whether or not pumas selectively preyed on radiocollared versus uncollared bighorn sheep (because marked animals are more conspicuous), and to examine movement patterns at and around bighorn sheep kill sites.

## Methods

### Study area and animals

The Peninsular Ranges of southern California extend approximately 150 km north from the United States-Mexico border. Lower elevations of the Peninsular Ranges are in the Colorado subdivision of the Sonoran Desert, and hot dry summers and mild winters characterize the climate [4]. Bighorn sheep in the Peninsular Ranges are a California protected species and have been federally listed as endangered since 1998 [5]. They are typically found below 1,400-m elevations in the eastern portion of the Peninsular Ranges, and are sympatric with mule deer (*Odocoileus hemionus*) at the upper elevations of bighorn sheep range. Our study from 2002-2004 included radiocollared and uncollared bighorn sheep from 5 of the 8 recognized subpopulations [6], and we use the term "radiocollar" to include either VHF (Telonics, Inc., Tempe, Arizona) or GPS (Televilt Simplex P-1D, Telemetry Solutions, Concord, California) collars on bighorn sheep. We placed GPS radiocollars on 3 pumas that each killed multiple bighorn sheep in the Peninsular Ranges: female F7 and her offspring males M5 and M6. During the entire time that M5 and M6 were GPS radiocollared for this study, they were independent of F7, and each hunted alone to feed only themselves. Puma GPS collars were programmed to acquire locations 4 times within a 24-h period: at night (0000 h), crepuscular periods (0600 and 1900 h), and midday (1200 h).

### Bighorn Sheep Population Estimates

The total number of bighorn sheep in each of the 5 subpopulations was estimated based on biennial helicopter surveys conducted by the California Department of Fish and Game. Using radiocollared bighorn sheep in each subpopulation as "marked" animals (each was also ear-tagged), estimates were calculated with capture-recapture methods using Chapman's [7] derivation of the Lincoln-Petersen estimator as described in Rubin et al. [6]. We subtracted the numbers of radiocollared sheep from the total subpopulation estimates to determine the numbers of uncollared bighorn sheep at risk in each subpopulation.

### Predation Events

Kill sites of radiocollared bighorn sheep were identified by field investigation of all radiocollars detected in mortality mode. For uncollared bighorn sheep, we identified potential kill sites by visually examining GPS data of radiocollared pumas for locations that suggested a particular puma was returning to or remaining at a kill site. When a dead bighorn sheep was found in the field, puma predation was ruled in or out as the cause of death following the criteria of Hayes et al. [8]. We subsequently developed an algorithm to identify potential kill sites with puma GPS data using a method similar to that of Anderson and Lindzey [9]. GPS data clusters representing potential kill sites were defined as 2 or more consecutive GPS locations at night or crepuscular times (1900, 0000, 0600 h) that occurred within 24 h and within 200 m of each other. The algorithm was then applied retrospectively to the GPS data from the 3 pumas to see if it delineated the 23 known bighorn sheep kill sites that we investigated.

We assigned puma GPS locations to 1 of 3 timeframes to facilitate statistical comparisons of how far pumas were located from kill sites in the periods before, during, and after a kill. The "during kill" timeframe was defined as the mean plus 1 standard deviation of the total time in hours that pumas spent at kills (1st arrival to final departure). The "before kill" and "after kill" timeframes were defined by adding this same length of time to the period before or after the "during kill" timeframe, respectively. We calculated the 2-dimensional Euclidean distances between puma locations and bighorn sheep kill sites using Universal Transverse Mercator (UTM) coordinates for GPS positions.

### Statistical Analysis

We tested the null hypothesis that there was no difference in predation by the 3 pumas on radiocollared versus uncollared bighorn sheep using the 2-sided Fisher's Exact Test (JMP Version 8.0, SAS Institute Inc., 2008). Our comparison was stratified by individual puma and geographical area because individual pumas may differ in their predilection for attacking radiocollared versus uncollared sheep, and geographical areas may influence the effect of radiocollars on risk of predation due to differences in cover, forage quality, or behaviour of sheep subpopulations, etc. We deemed results significant when *P *< 0.05. Assuming equal expected kill frequencies (0.25) at each of the 4 times of day, we compared estimated and expected times of death using an exact multinomial test [10].

We tested the null hypotheses that pumas were not found at different distances from kills across the 3 timeframes, and that no differences existed in distances among the 4 different times of day using mixed-models repeated-measures analysis of variance (ANOVA)[11]. When significant differences were detected, pairwise comparisons (randomized by puma) were made using the Tukey-Kramer HSD (honestly significant difference) test. We compared overall mean distances to kill sites between timeframes, and within timeframes we compared overall mean distances both across and among times of day. Finally, within the during-kill timeframe, distances were evaluated by time of day and sequential day; mean daily distances to kills sites were compared using mixed-models repeated-measures ANOVA with the Tukey-Kramer HSD test. All statistical tests except the exact multinomial test were conducted using JMP software (Version 8.0, SAS Institute Inc., 2008).

## Results

### Predation on bighorn sheep

**Figure 1:**
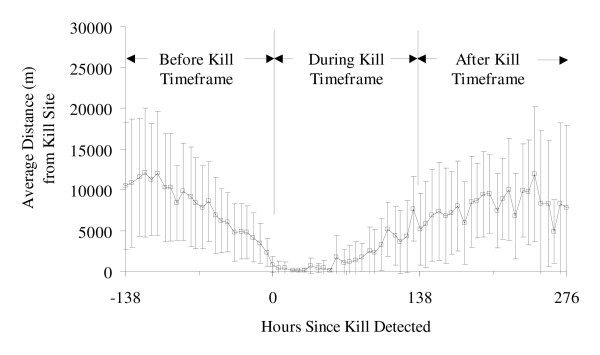
**Average distance of pumas from bighorn sheep kill sites as a function of time**. Average distance of pumas F7, M5, and M6 from 22 bighorn sheep kill sites as a function of time in the Peninsular Ranges of southern California, USA, January 2002-September 2004. Error bars show standard deviation of distance. Data are normalized to time of kill detection (*t *= 0) for comparison. The mean plus 1 standard deviation of time spent at a kill site was found in this study to be 138 h (5.7 days). This was used to bound the before, during and after kill timeframe analyses for comparison.

The 3 pumas killed 23 bighorn sheep over the course of the study, but they did not (P > 0.05) preferentially prey on marked (radiocollared and ear-tagged) versus unmarked bighorn sheep (Table 1). Predation occurred primarily during crepuscular and night time hours, and 22 kill sites were identified by the occurrence of 2 or more consecutive puma GPS locations within 200 m of each other at 1900, 0000, and 0600 h (Table 2). The overall mean time spent at kill sites was 92 h with a standard deviation of 46 h. The during-kill timeframe, defined as the mean time spent at kill sites plus 1 standard deviation, was determined to be 138 h (Fig. 1). Therefore, the before-kill period corresponded to the 138 h period before the kill was detected (days 1-6 pre-kill), and the after-kill timeframe corresponded to 138-276 h after detection (days 7-12 post-kill). Expected and estimated kill frequencies at different times of day differed and no kills were found to occur at 1200 h (*P *= 0.043). Pumas were at similar distances from kill sites at different times of day in both the before-kill and after-kill time periods (Table 3, *P *= 0.884 and *P *= 0.658, respectively). However, in the during-kill timeframe, pumas were significantly farther from kill sites at midday (1200 h versus 0600 h), presumably at day-bed sites (Table 3; *P *= 0.044). More detailed examination showed that pumas were very close to kill sites during days 1-3 post-kill and progressively farther away during days 4-6 post-kill (Table 4; *P *< 0.0001).

**Table 1:** Ratio of radiocollared and uncollared bighorn sheep killed by pumas

Bighorn Sheep Subpopulation	Puma	Killed RCB/UCB^a^	Exposed RCB/UCB^a^	P Value
Coyote Canyon	F7		10/25	0.292
North San Ysidro Mountains	F7	0/2	11/36	1.0
South San Ysidro Mountains^c^	F7	2/3	13/28	0.645
Vallecito Mountains^c^	F7	-	-	N/A
Carrizo Canyon^c^	F7	-	-	N/A
Total	F7	2/10	34/89	0.508

Coyote Canyon	M5	0/1	8/27	1.0
North San Ysidro Mountains	M5	0/0	9/38	1.0
South San Ysidro Mountains^c^	M5	0/3	12/29	0.543
Vallecito Mountains^c^	M5	0/0	12/143	1.0
Carrizo Canyon^c^	M5	0/2	9/118	1.0
Total	M5	0/6	50/355	1.0

Coyote Canyon	M6	1^b^/1	9/38	0.35
North San Ysidro Mountains	M6	1/0	6/44	0.12
South San Ysidro Mountains^c^	M6	-	-	N/A
Vallecito Mountains^c^	M6	0/2	14/136	1.0
Carrizo Canyon^c^	M6	-	-	N/A
Total	M6	2/3	29/218	0.106

Ratio of radiocollared and uncollared bighorn sheep killed by pumas (M5, M6, and F7) by subpopulation in the Peninsular Ranges of southern California, USA, January 2002-September 2004.

^a ^RCB = radiocollared bighorn sheep, UCB = uncollared bighorn sheep. P values shown are 2-sided Fisher's Exact Test values comparing RCB and UCB predation.

^b ^A radiocollared sheep was killed on 7 May 2004. Due to a collar data transmission error from 1 March to 25 May 2004, this kill could not be definitively linked to M6 with the use of GPS data. M6 passed near this kill site in this approximate timeframe and within 928 m of the site on 20 June 2004, based on VHF tracking and GPS data, respectively. This kill was conservatively attributed to M6.

^c ^Blank cells denote subpopulations through which the puma was not known to hunt or pass.

**Table 2:** Number of GPS location clusters identified and kills located by Pumas

Puma	Total # of Location Clusters Identified^a^	Total # of Kills Located	% of Kills w/in Clusters
F7 (Jan 2002-Feb 2003)	94	12	100
M5 (Nov 2002-Jul 2003)	27	6	100
M6 (Nov 2003-Sep 2004)	20	4^b^	100^b^

Total	141	22	

Number of GPS location clusters and bighorn sheep kill sites identified for 3 pumas in the Peninsular Ranges of southern California, USA, January 2002-September 2004. Clusters were defined as consecutive GPS location acquisitions within 24 hours and 200 m of each other.

^a ^Due to logistical constraints, not all GPS location clusters were investigated for bighorn sheep kills. Additionally, some clusters corresponded to other species like deer so no direct comparison was made.

^b ^A radiocollared sheep was killed on 7 May 2004. Due to a collar data transmission error from 1 March to 25 May 2004, this kill could not be definitively linked to M6 with the use of GPS data. M6 passed near this kill site in this approximate timeframe and within 928 m of the site on 20 June 2004, based on VHF tracking and GPS data, respectively. This kill was conservatively attributed to M6.

**Table 3:** Puma distance from kill sites across four times of day

		Distance from kill site (m)			
Timeframe^a^	0000 h	0600 h	1200 h	1900 h	Overall
	Median [range]	Median [range]	Median [range]	Median [range]	Median [range]
Before kill	5880 [44-27500]	6520 [380-31890]	6330 [21-30930]	5960 [20-29280]	6090 [20-31890]
During kill	44^c^ [4-13560]	68^b, c^ [1-10590]	1240^b, c^ [5-11780]	430^c^ [1-11580]	113^c^ [1-13560]
After kill	8060 [8-22730]	8830 [2-27640]	8020 [140-27760]	6950 [20-22200]	8020 [2-27760]

Comparison of median and range of distance (m) of 3 pumas from known bighorn sheep kill sites across 4 times of day (0000, 0600, 1200, and 1900 h) and 3 timeframes (before kill, during kill and after kill) in the Peninsular Ranges of southern California, USA, January 2002-September 2004.

^a ^The mean plus 1 standard deviation of time spent at a kill site was found in this study to be 138 hours (5.7 days). This was used to bound the before, during and after kill timeframe analyses for comparison. The distances from kill sites at different times of day in the before (F = 0.217, df = 3, *P *= 0.884) and after (F = 0.536, df = 3, *P *= 0.658) kill timeframes did not differ significantly.

^b ^The distances from kill sites at 1200 h in the during kill timeframe was significantly larger (F = 2.73, df = 3, *P *= 0.044) than distances at 0600 h. The distances at 0000 h and 1900 h did not differ from other times.

^c ^Pumas were significantly closer (F = 152.4, df = 2, *P *< 0.0001) to kill sites in the during kill timeframe than in the other timeframes.

**Table 4:** Puma distance from bighorn sheep kill sites

	Distance from kill site (m)					
	Day 1	Day 2	Day 3	Day 4	Day 5	Day 6
	Median	Median	Median	Median	Median	Median
Time^a^	[range]	[range]	[range]	[range]	[range]	[range]
0000 h	29	15	35	23	1420	2480
	[14-150]	[5-430]	[5-3660]	[4-6090]	[6-10010]	[8-9520]
0600 h	16	24	18	195	3050	2710
	[1-170]	[3-1040]	[7-670]	[8-9250]	[15-8900]	[6-10590]
1200 h	40	163	360	1340	5790	8050
	[5-450]	[23-2480]	[7-7770]	[8-5880]	[1000-10470]	[2610-11780]
1900 h	17	46	172	1470	2670	4480
	[1-260]	[5-1870]	[4-5420]	[5-10910]	[19-10680]	[4-11580]
Overall^b^	26^A^	30^A^	53^A, B^	740^B^	2950^C^	3200^D^
	[1-450]	[3-2480]	[4-7770]	[4-10910]	[6-10680]	[4-11780]

Comparison of median distances (m) of 3 pumas from 22 bighorn sheep kill sites by time of day for the six days following a kill. Study was performed in the Peninsular Ranges of southern California, USA, January 2002-September 2004.

^a ^The times shown are actual times of day (GMT-7), not hours since kill. Since not all kills occurred at midnight on the 1st day, there are less data points in some of the earlier hour's classes on the 1st day.

^b ^Days with overall means not sharing the same capital letter superscript (A, B, C, D) are significantly (F = 44.2, df = 5, *P *< 0.0001) different.

## Discussion

Although deer are the primary prey of pumas in North America, pumas can be an important cause of mortality for bighorn sheep [1,8,12-15]. Radiocollars have been used extensively to study bighorn sheep, and a valid concern is whether or not radiocollars or other auxiliary markings such as ear tags place bighorn sheep at increased risk of predation. We tested the "conspicuous individual hypothesis" [16] and found that there was no difference in puma predation upon radiocollared and uncollared bighorn sheep in the Peninsular Ranges (Table 1).

This study was not intended to evaluate survival and cause-specific mortality among bighorn sheep, but our results indicate that puma predation rates on radiocollared bighorn sheep can be used as an index to estimate predation rates on uncollared bighorn sheep in the same population. For example, Hayes et al. [8] found that annual adult mortality rates due to predation ranged from 0.08 to 0.25 among radiocollared bighorn sheep in our study area from 1992-1998. Our results indicate that puma predation was responsible for a similar cause-specific mortality rates among non-radiocollared adult bighorn sheep during the same time period. Our findings have important implications for other studies where survival and predation-specific mortality rates are based on data from radiocollared bighorn sheep [16].

One potential caveat to our predation results is that pumas have been shown to scavenge deer carcasses found in favorable locations (i.e., along puma travel routes) at higher elevations (where cooler temperatures reduce spoilage) in the Peninsular Ranges [17]. We consider puma scavenging to be an unlikely event in the desert environment where bighorn sheep are relatively uncommon, widely dispersed, and where conditions rapidly spoil carcasses. Regardless, it is clear that radiocollars did not put bighorn sheep at increased risk of predation by the 3 pumas studied here (acknowledging that only a small number of pumas were studied).

The movements of pumas before, during and after killing bighorn sheep are poorly understood relative to what is known about puma predation on deer, and our delineation of timeframes allowed quantitative comparisons of the sedentary and travelling behaviours of pumas across kills and individuals. Pumas were significantly closer to kills in the 6-day during-kill timeframe than in either the before-kill or after-kill timeframes (Table 2; Fig. 1), consistent with the view of pumas as wide ranging, opportunistic predators of bighorn sheep. The majority of bighorn sheep kills occurred during night and early morning (0000, 0600 h), similar to timing of puma predation on deer [18]. Pumas mostly fed on kills during the night and were much more likely to be detected at kill sites between dawn and dusk (Tables 3 and 4) than during the daylight hours when they move to day bed sites [2]. We encourage researchers to exploit these predictable behaviours and identify puma kill sites and investigate prey selection by designing studies that determine whether puma locations are spatially clustered between dusk and dawn.

## Conclusion

We tested the "conspicuous individual hypothesis" and found that there was no difference in puma predation upon radiocollared and uncollared bighorn sheep. Our results are consistent with the view of pumas as wide ranging predators of desert bighorn sheep (and other prey species) that tend to cover substantial distances both before and after making kills. Researchers can exploit predictable post-kill behaviours to identify puma kill sites and investigate prey selection by designing studies that detect clusters of locations between dusk and dawn.

## Competing interests

The authors declare that they have no competing interests.

## Authors' contributions

SC participated in study design, conducted analyses, and drafted the manuscript. ER, RB, and WB conceived the study, conducted field research, and revised the manuscript. CK assisted with statistical analyses and manuscript review and revision. This manuscript was prepared as part of SC's dissertation research under the supervision of WB. All authors have approved the final manuscript.

## Authors' informations

WB and ER were members of the USFWS Recovery Team for Peninsular Bighorn Sheep during this study, and RB was the CDFG biologist responsible for the study area. All research was conducted with appropriate state and federal permits. Responsible agencies and managers were kept apprised of puma behavior and immediately notified of predation events throughout the study.
